# Quantitative Electroencephalographic Measures of Voltage Amplitude and Dominant Frequency Associated With the Stroop Color-Conflict Cognitive-Interference Task in Medical Students

**DOI:** 10.7759/cureus.86277

**Published:** 2025-06-18

**Authors:** Darine Alame, Merin Chandanathil, Brianna Easton, Shweta Verma, N'Kozi Bennett, Nicole R Moldovan, Denise Crowley, Richard M Millis

**Affiliations:** 1 Department of Physiology, American University of Antigua, St. John’s, ATG; 2 Department of Occupational Therapy, Monmouth University, West Long Branch, USA

**Keywords:** dominant frequency, medical school students, quantitative eeg (qeeg), stroop test, voltage amplitude

## Abstract

Background

Cognitive load theory postulates that effective learning depends on balancing a learner's cognitive capacity with cognitive load. Medical students are required to answer complex multiple-choice questions (MCQs) that involve complex vignettes and distractors, in 90 s per question. This demands the ability to rapidly process information, filter out irrelevant data, and suppress incorrect yet tempting answer choices. The Stroop color-conflict test represents a cognitive interference task that may simulate time-limited conditions for answering MCQs. This exploratory study tested whether selected quantitative electroencephalographic (qEEG) indices could behave as biomarkers that remain stable across sequential Stroop loads.

Methods

Thirteen healthy adults (11 retained after outlier removal) completed a midline (Fz, Cz, Pz) qEEG protocol comprising (i) 5 minutes of resting baseline, (ii) 5 minutes after a congruent low-load (LL) Stroop test and (iii) 5 minutes after an incongruent high-load (HL) Stroop test. Voltage amplitude (µV) and mode frequency (Hz) were extracted for theta (4-7 Hz), alpha (8-12 Hz), low-beta (12-20 Hz) and high-beta (20-30 Hz) bandwidths. Derived ratios, θ/β, α/β, θ/α and high-β/low-β, plus a frontal-posterior theta ratio (Fz/Pz), were analyzed with paired t-tests and repeated-measures ANOVA. Outliers were removed using a strict |z| > 2 threshold applied to every site-specific metric.

Results

Significant Baseline → LL load sensitivity was found for alpha-dominant (mode) frequency. The dominant frequencies, voltage amplitudes and voltage amplitude ratios for the other bandwidths (θ, low-β, high-β) were nonsignificant and therefore not load sensitive. None of the markers exhibited significant changes from LL → HL. Alpha voltage amplitudes were found to be higher at Pz than at Cz and Fz, exhibiting posterior dominant site sensitivity. High-β/low-β and θ/β ratios were found to be higher at Fz and Cz than at Pz, exhibiting frontal dominant site sensitivity.

Conclusion

These findings suggest significant Stroop testing-related qEEG changes in medical students trained to answer complex MCQs under time constraints. Alpha dominant frequency was found to be load sensitive but site insensitive. Load insensitivity of alpha voltage amplitude, θ/β ratio and high-β/low-β ratio at the Cz, Fz and Pz midline recording sites suggests site specificity of these variables. These findings appear to support the hypothesis that the site-specific topographic markers alpha voltage amplitude, θ/β and high-β/low-β ratio may be useful for characterizing responses to Stroop testing. However, the load sensitivity of alpha dominant frequency measured at the Cz, Fz and Pz midline recording sites may be useful for workload tracking to identify and remediate information-processing problems. These preliminary findings should be interpreted cautiously pending larger studies of cognitive loading in other populations of learners trained to take high-stakes, time-limited examinations.

## Introduction

Quantitative electroencephalography (qEEG) is increasingly being used to study how the brain responds to mental tasks because it can measure brain activity in real time [1]. Research has shown that certain brainwave patterns, especially those in the alpha frequency range, are linked to key mental functions like attention and working memory, which are major parts of cognitive load [2]. Both the strength (amplitude) and speed (frequency) of alpha waves have been found to change depending on how difficult a task is and how much mental effort it requires [3-5].

This makes qEEG a promising tool for measuring cognitive load, especially in educational and training settings, because it provides continuous feedback about how hard the brain is working during a task. Unlike traditional methods of assessing mental effort, qEEG can track changes in cognitive load as they happen. Studies have shown that changes in alpha wave activity across different parts of the brain reflect how learners respond to tasks of varying difficulty [6-9]. This kind of real-time brain data can help educators adjust the complexity of learning activities to match students' mental capacity, potentially improving attention, learning, and performance [10-13].

In this study, we aimed to use qEEG to explore how medical students respond to mental interference while solving timed, multiple-choice questions (MCQs) based on clinical scenarios. These types of questions require students to quickly identify relevant information, ignore distractions, and use executive thinking skills, much like what is needed to succeed in the Stroop color-word interference test. Our goal was to determine whether qEEG can detect differences in brain activity related to this kind of cognitive challenge, especially in the frontal and parietal regions of the brain, which are associated with attention and decision-making.

Poor performance on high-level MCQs is a major cause of academic difficulty in medical school [14], and test anxiety can worsen performance by interfering with working memory [15]. By using qEEG, we hope to identify reliable markers, such as alpha frequency, to discover how students process and manage cognitive load under pressure, with the long-term goal of improving test preparation and educational outcomes [16].

The Stroop task was used because it provides a well-established way to create and measure cognitive interference. When the color and word match (congruent trials), the brain is under minimal strain. When they don’t match (incongruent trials), the brain must work harder to suppress automatic responses and engage executive control, which is known to increase brain activity in the prefrontal cortex - especially in the beta frequency band [17].

The present study focuses on how qEEG activity changes in response to low-load (LL) and high-load (HL) Stroop trials. Specifically, we examined whether certain brain activity measures are sensitive to cognitive load (i.e., they change from LL to HL trials) and whether they are sensitive to recording site (i.e., they vary between the frontal, central, and parietal midline brain areas). We also analyzed common EEG ratio metrics such as θ/β, α/β, θ/α and high-β/low-β ratios, which are often linked to attentional control [18, 19]. The main objective is to identify which of these measures best reflect how cognitive load is processed across different brain regions. This exploratory study tested whether selected qEEG indices could behave as biomarkers that remain stable across sequential Stroop loads.

## Materials and methods

Participants

Male and female year 1 (Y1) and year 2 (Y2) medical students enrolled in the preclinical basic science phase of our institution's medical curriculum were recruited by a student-faculty team of researchers at our biannual research interest group and academic club fair. Each participant signed an Institutional Review Board (IRB) approved Informed Consent document and questionnaire providing demographic information and a list of clinical diagnoses and medications. Seven Y1 and six Y2 students were selected as participants based on the absence of significant neuropsychiatric disease, potentially interfering medication regimens, availability and willingness to adhere to the pre-study protocol of overnight fasting, abstaining from food until the study sessions, medications and hair washing to reduce the likelihood of cosmetic-induced recording artifacts. Thirteen preclinical medical students were studied in the morning between 8:00 AM and 12:00 PM, after overnight fasting and refraining from nicotine, alcohol, caffeine and marijuana-related products for the prior 12 hours.

Quantitative encephalography

Resting‐state EEG was recorded with a 19-channel international 10-20 montage using a BrainMaster Discovery 40 amplifier (BrainMaster Technologies, Bedford, OH, USA). Signals were referenced to linked ears (A1/A2), digitized at 24 bit, 512 Hz, and filtered online with a 0.5 Hz high-pass, 70 Hz low-pass and 60 Hz notch filter; electrode impedances were kept below 10 kΩ. Continuous data were auto-segmented into contiguous 1-s epochs. Epochs exceeding ±100 µV peak-to-peak or showing excessive 24-40 Hz (muscle) or 55-65 Hz (line) power were rejected automatically. Artifact-free epochs were analyzed in a qEEG-based brain mapping editor v2025-04-20 system (NewMind Technologies, Roswell, GA, USA). Welch-averaged periodograms were computed (2-s Hamming window, 50% overlap, 1024-point FFT). Band-limited power was integrated in canonical frequency bands and converted to root-mean-square voltage (µV), which was exported in comma-separated value (CSV) format for statistical analysis. EEG recordings (19 monopolar 10-20 channels, A/D=256 Hz, 24 bit) were uploaded to New Mind Maps Analysis Suite (v2025-04-20, NewMind Technologies, Roswell, GA, USA). Continuous data were auto-segmented into 1-s epochs. Epochs presenting peak-to-peak amplitudes > ±100 µV, baseline drifts > 50 µV s-¹, or point-wise deviations > 5 SD were rejected; remaining epochs were also discarded when 24-40 Hz or 55-65 Hz power exceeded 2.5 SD of the running mean.

Averaged power spectral densities (PSDs) were integrated within the delta (1-4 Hz), theta (4-8 Hz), alpha (8-12 Hz), low-beta (12-15 Hz), beta (15-25 Hz), high-beta (25-30 Hz) and gamma (30-40 Hz) bands. The square root of the integrated power was reported as root-mean-square (RMS) voltage (µV). These µV magnitudes, along with dominant frequency, coherence, phase and asymmetry metrics, were exported in CSV format for subsequent statistical analysis. The use of RMS amplitudes, rather than log-transformed power, preserves linear scaling and facilitates comparison with normative qEEG atlases.

QEEG recordings were performed using electrode caps containing 19 electrodes situated according to the international 10-20 system and calibrated to standard electrical impedances. QEEG recordings were made with the subjects seated upright in a comfortable position with eyes closed immediately before and after exposure to baseline control, LL and HL conditions. Measurements of voltage amplitude and dominant mode frequency were analyzed for the standard frequency bands of theta (4-7 Hz), alpha (8-12 Hz), low-beta (13-18 Hz), beta (13-30 Hz) and high-beta (20-30 Hz). The delta (1-3 Hz) and gamma (> 30 Hz) bandwidths were omitted from the analysis because of concerns about stationarity and signal-to-noise ratio. QEEG recording artifacts were removed by a combination of manual and electronic editing (NewMind Maps Technologies, Inc., Roswell, GA, USA). The present study was confined to evaluating midline voltage amplitude and dominant (mode) frequency at the frontal (Fz), central (Cz) and parietal (Pz) electrodes. The qEEG recordings were analyzed every 30 s for 5 min immediately before (baseline control) and exposure to two successive periods of Stroop color-conflict testing with a 5-min recording between the two periods.

Stroop protocol

The Stroop protocol consisted of 40 trials per block (20 congruent, 20 incongruent, random order); stimulus 500 ms, inter-stimulus interval (ISI) 1000 ms; responses verbalized; E-Prime 2.0 (Psychology Software Tools, Inc., Pittsburgh, PA, USA) used for stimulus delivery.

Because overt button‑presses and articulatory movements during the Stroop task produced non‑stationary artifacts in preliminary recordings, qEEG was obtained for 5 min immediately before (Pre) and after (Post) the dual‑trial Stroop sequence while subjects rested with eyes closed. Each 5‑min record was partitioned into ten non‑overlapping 30‑s epochs, the minimum length that yields reliable power‑spectral density estimates for the 4-30 Hz range studied. Validity of EEG recording 3 min after two memory tasks demonstrated that α-band changes at 5 min post-task still tracked the cognitive effort invested during the preceding task [20]. For every subject and epoch, we extracted mean voltage amplitude (µV) and dominant frequency (Hz) for each bandwidth. Outcomes were then averaged across the ten epochs to produce a pretest and posttest value per participant. This approach (i) eliminated movement and speech artifacts intrinsic to task execution, (ii) captured tonic neurophysiological after‑effects of interference resolution that are reported to persist up to one minute, and (iii) increased within‑subject spectral reliability by reducing the standard errors.

Statistical analysis

The Stroop color-conflict test involved participants describing the color of a word presented at a computer-controlled pace, rather than reading the word itself. The test consisted of two trials: in the first (congruent) trial, the color matched the word, whereas in the second (incongruent) trial, the color did not match the word. Editing of the data was done as follows: Site × Bandwidth × Metric (voltage amplitude, frequency, ratio) we z-scored across subjects; |z| > 2 was the criterion for exclusion. The rationale for this method is that extreme points have disproportionately high leverage on averages and, in qEEG practice, are far more likely to arise from transient artifacts (muscle bursts, electrode pops, sudden movement) than from true neurophysiological variance. Trimming observations whose |z| > 2 preserves > 95% of the data, avoiding undue loss of statistical power and removes the small proportion of samples most likely to distort parameter estimates and inflate error variance. This exact cut-off has long been used in normative-database construction, where z-scores outside ±2 SD are treated as statistically atypical and excluded from the reference set [21]. Accordingly, applying a |z| > 2 rule in the present dataset removes only the most extreme 4-5% of samples, improving the robustness of all downstream qEEG metrics while leaving the genuine neurocognitive signal essentially intact. After data cleaning, we retained 8 observations for the Baseline ↔ LL comparison and 6 observations for the LL ↔ HL comparison. Paired two-tailed t-tests compared Baseline vs LL and LL vs HL; effect size (d). Post-hoc power computation was based on Cohen’s d and standard deviation (α=0.05). Trait stability was cross-checked with repeated-measures ANOVAs (Site × Condition).

This study was a preliminary, exploratory investigation with a small sample (13 recruited, 11 analyzed after outlier removal) and no prior data to estimate effect sizes. Consequently, a formal a priori power calculation was not feasible. Instead, we assessed statistical power retrospectively (post hoc) using the observed effect sizes (Cohen’s d) from our repeated-measures ANOVA and paired t-test comparisons, employing standard power analysis procedures [22, 23]. This approach is justified in exploratory EEG research where effect sizes are initially unknown, as it provides a gauge of the achieved power to detect effects and informs future study planning [24]. Our post hoc power analysis indicated that the within-subject design was reasonably sensitive to moderate-to-large effects: for example, observed effects in the moderate range (Cohen’s d=0.5-0.6) correspond to roughly 50-60% power, whereas large effects (d=0.8-1.0) correspond to approximately 65-80% power at α=0.05 [25]. Thus, only large effects would be detectable with conventional power (1-β = 0.8) given our sample size [24], while smaller effects likely went undetected. We report these post hoc power estimates to demonstrate the reliability of our significant findings and acknowledge the limits of a “small-N” design. This retrospective power analysis should be interpreted cautiously pending a larger study [25]. This approach is appropriate for a preliminary study and aligns with recommendations for analyzing pilot qEEG data in the absence of prior effect size information [24]. Table 1 summarizes the paired Cohen’s d and observed power (1-β) for the relevant qEEG metrics (alpha power, theta-beta ratio, high-beta/low-beta ratio, and alpha dominant frequency) at the midline frontal (Fz), central (Cz) and parietal (Pz) qEEG electrode recording sites studied.

**Table 1:** Post-hoc power analysis of qEEG changes. Comparisons are Baseline vs Low Load (LL), LL vs High Load (HL), and Baseline vs HL. Power was calculated for paired t-tests at α=0.05 (two-tailed) based on the observed effect size (Cohen’s d) and N=11. Effect sizes (d) calculated as the mean within-subject difference divided by the standard deviation of differences (paired d). Observed power (1–β) computed using the above d and sample size by standard paired t-test (two-tailed, α=0.05). Note: all comparisons show low observed power (well below the conventional 0.80 threshold), reflecting the small sample and modest effect sizes. Only the Pz alpha dominant frequency showed a large effect (d=1.0) with high post-hoc power (>0.90). These post-hoc power estimates should be interpreted with caution, as they are based on observed data and may not reflect true effect sizes – especially with a small sample, the estimates (and thus calculated power) are uncertain.

Metric	Site	d (Baseline vs LL)	Power (Baseline vs LL)	d (LL vs HL)	Power (LL vs HL)	d (Baseline vs HL)	Power (Baseline vs HL)
Alpha power	Fz	0.08	0.06	0.13	0.07	0.10	0.06
	Cz	–0.10	0.06	0.23	0.12	0.16	0.08
	Pz	–0.04	0.05	0.29	0.17	0.14	0.07
Theta–beta ratio	Fz	–0.28	0.16	–0.34	0.20	–0.34	0.21
	Cz	–0.53	0.42	0.06	0.05	–0.40	0.26
	Pz	–0.40	0.26	0.27	0.15	–0.29	0.16
High-beta/low-beta ratio	Fz	–0.10	0.06	0.22	0.12	–0.01	0.05
	Cz	0.01	0.05	–0.08	0.06	–0.05	0.05
	Pz	–0.24	0.13	0.13	0.07	–0.19	0.10
Alpha dominant frequency	Fz	0.58	0.49	–0.23	0.12	0.48	0.36
	Cz	0.37	0.24	–0.16	0.08	0.23	0.12
	Pz	1.23	0.98	–0.08	0.06	0.99	0.90

## Results

Table 2 summarizes the characteristics of the study group. 

**Table 2:** Characteristics of the study group.

Characteristic	Mean ± SD
Age	27.8 ± 7.8
Sex	6 male, 7 female
Handedness	12 right, 1 left
Theta-beta ratio	1.78 ± 1.21

Baseline → Low-load (LL) conditions

**Figure 1:**
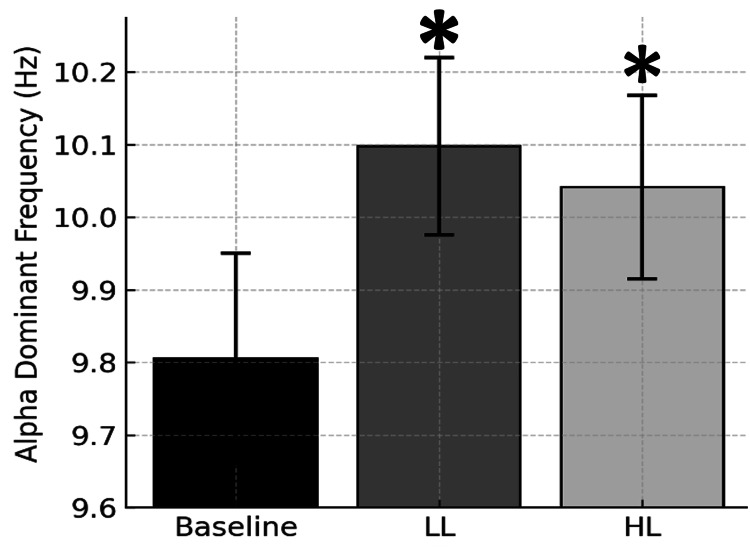
Alpha dominant frequency across conditions. Bars represent site-averaged mean (± SEM) alpha dominant (mode) frequencies, expressed in Hz, measured by qEEG for baseline control and for 5 minutes immediately after congruent Stroop test low cognitive load (LL) and noncongruent Stroop test high cognitive load (HL) conditions. *Significantly different than baseline, p < 0.05.

Figure 1 shows the site-averaged Stroop load-induced changes in alpha dominant (mode) frequencies. A load-sensitive increase was observed after exposure to the LL Stroop test; the change in alpha dominant frequency from the LL to the HL Stroop test was not significant. This finding suggests alpha dominant frequency sensitivity to Stroop-induced loading.

Stroop Baseline →LL changes in other bandwidth dominant frequencies (θ, β), voltage amplitudes (θ, α, β) and voltage amplitude ratios were not significant (p > 0.05).

Low-load (LL) → High-load (HL) conditions

Stroop LL→HL changes in the voltage amplitudes, mode frequencies and voltage amplitude ratios studied were not significant (p > 0.05).

Topographical findings

**Figure 2:**
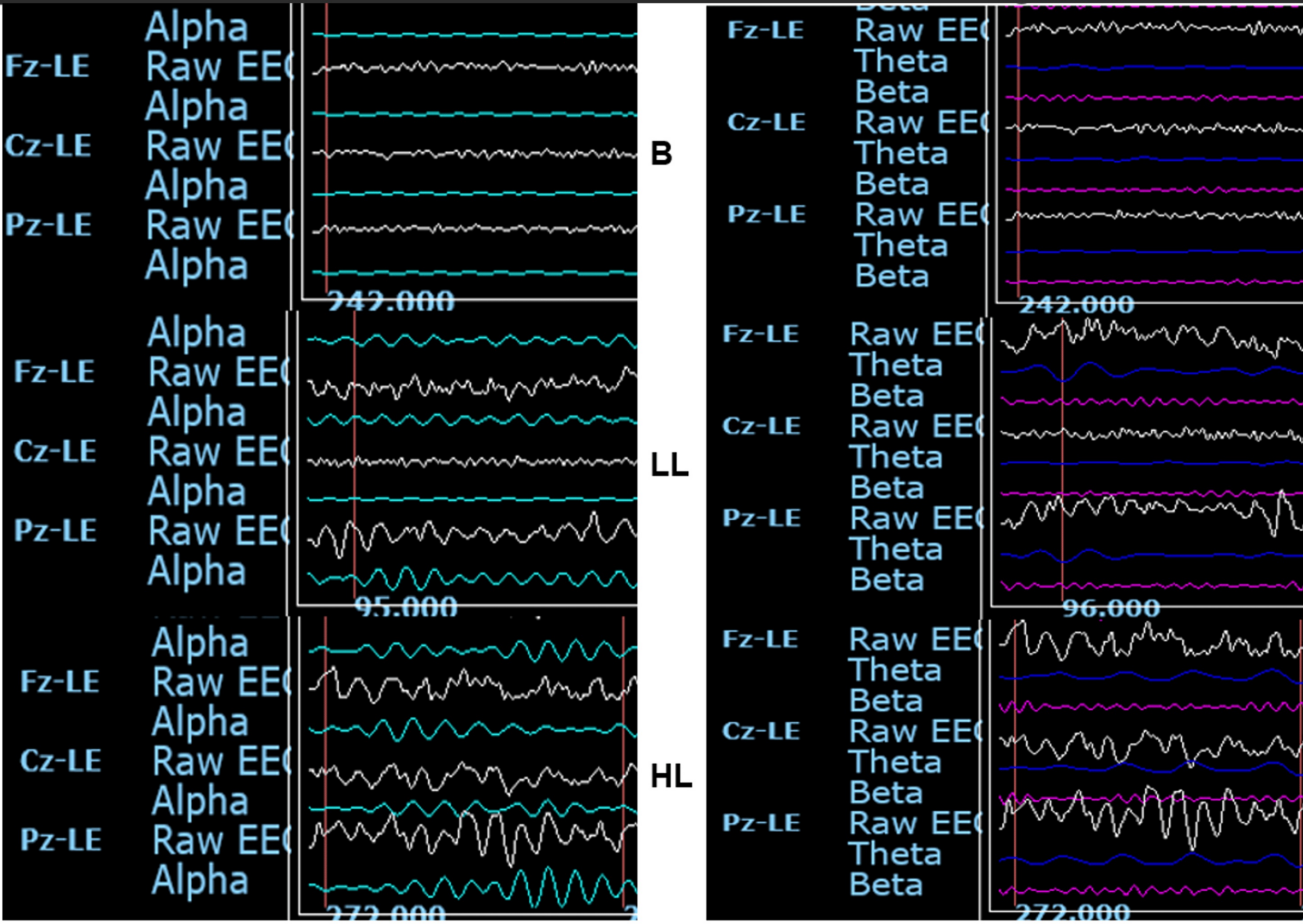
Representative qEEG tracings. Panels show one-second qEEG recordings from a 27-year-old male study participant in the same time frames. qEEG tracings are for baseline control (B) and for 5 min immediately after congruent Stroop test low cognitive load (LL) and noncongruent Stroop test high cognitive load (HL) conditions. Left panel: Raw left ear (LE) referenced EEGs (white tracings) show the qEEG load-insensitive, site-sensitive changes in alpha (8-12 Hz) voltages (green tracings) at midline fontal (Fz-LE), central (Cz-LE) and parietal (Pz-LE) sites. Right panel: LE-referenced raw EEGs (white tracings) filtered to show the theta (4-7 Hz) voltages (blue tracings), and beta (13-30 Hz) voltages (pink tracings) at the same sites and loads, reflecting the data used to compute the theta/beta voltage amplitude ratios.

Figure 2 shows two panels of representative one-second raw EEG recordings from one participant, a 27-year-old male. One panel presents the raw EEGs filtered to show the qEEG load-insensitive, site-sensitive changes in alpha voltage amplitude at Fz, Cz and Pz, under baseline, LL and HL conditions. A second panel depicts the raw EEGs filtered to show the theta and beta voltage amplitudes at the same sites and loads, reflecting the tracings used to compute theta/beta voltage amplitude ratios.

**Figure 3:**
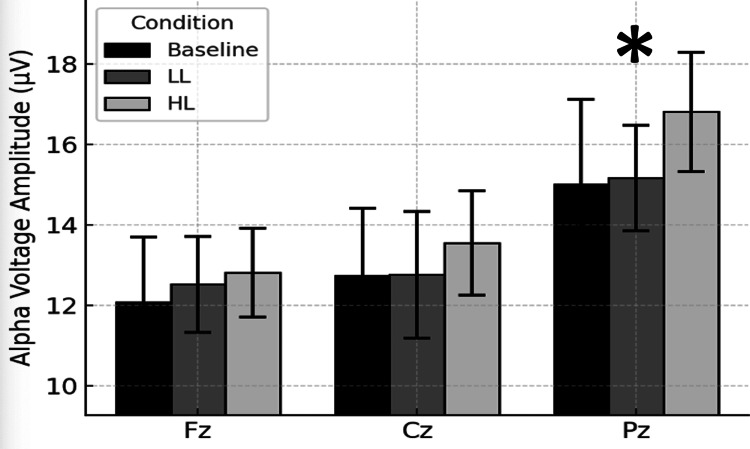
Alpha voltage amplitude across sites. Bars represent mean (± SEM) alpha voltage amplitudes measured by qEEG for baseline control and for 5 minutes immediately after congruent Stroop test low cognitive load (LL) and noncongruent Stroop test high cognitive load (HL) conditions. Measurements were at midline frontal (Fz), central (Cz) and parietal (Pz) electrodes. *Pz significantly different than Cz and Fz, p < 0.01.

Figure 3 depicts the site-specific alpha voltage amplitudes across the baseline, LL and HL Stroop testing conditions. The alpha voltage amplitudes recorded at Pz were significantly higher than those recorded at Fz and Cz. This finding suggests posterior cortical dominance of alpha voltage amplitude topography.

**Figure 4:**
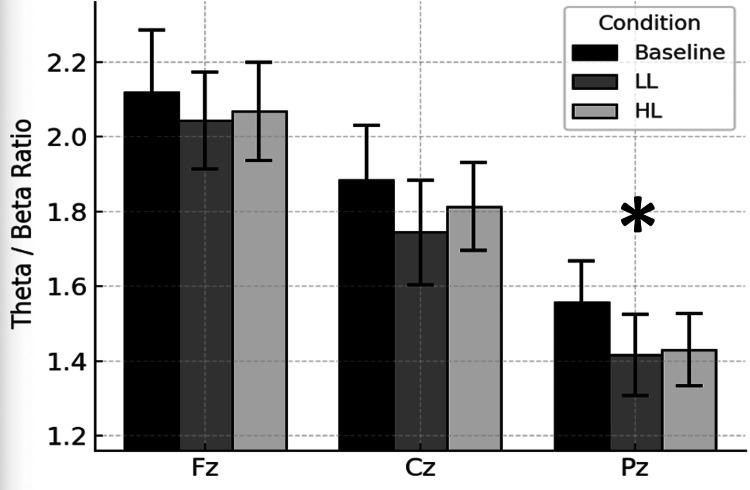
Theta-beta ratio across sites. Bars represent mean (± SEM) theta/beta voltage amplitude ratios computed from qEEG measurements for baseline control and for 5 minutes immediately after congruent Stroop test low cognitive load (LL) and noncongruent Stroop test high cognitive load (HL) conditions. Measurements were at midline frontal (Fz), central (Cz) and parietal (Pz) electrodes. *Pz significantly different than Cz and Fz, p < 0.05.

Figure 4 shows the site-specific θ/β ratios across the baseline, LL and HL Stroop testing conditions. The alpha θ/β ratios at Pz were significantly lower than those at Fz and Cz. This finding suggests frontal cortical dominance of θ/β ratio topography.

**Figure 5:**
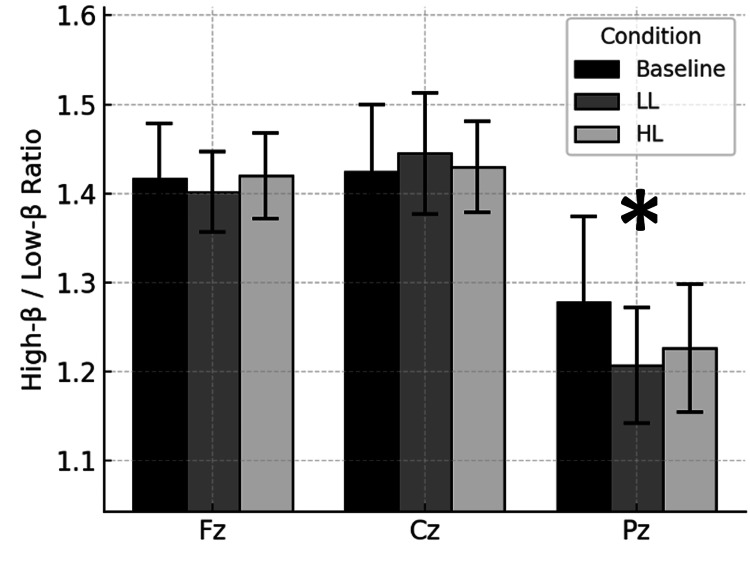
High-β/low-β ratio across sites. Bars represent mean (± SEM) high-β/low-β voltage amplitude ratios computed from qEEG measurements for baseline control and for 5 minutes immediately after congruent Stroop test low cognitive load (LL) and noncongruent Stroop test high cognitive load (HL) conditions. Measurements were at midline frontal (Fz), central (Cz) and parietal (Pz) electrodes. *Pz significantly different than Cz and Fz, p < 0.05.

Figure 5 presents the site-specific high-β/low-β ratios across the baseline, LL and HL Stroop testing conditions. The high-β/low-β ratios at Pz were also significantly lower than those at Fz and Cz. This finding suggests frontal cortical dominance of high-β/low-β ratio topography.

Across all conditions, Pz showed consistently lower θ/β and high-β/low-β ratios and higher alpha voltage amplitude at Pz than at either Cz or Fz. These spatial profiles did not interact with the Stroop-induced cognitive load condition, thereby suggesting a cognitive trait-like response.

## Discussion

Cognitive interference and executive function in the Stroop task

The Stroop color-conflict test is one of the most widely used paradigms for investigating cognitive interference and response inhibition [26]. The task presents participants with color words (e.g., "RED," "BLUE," "GREEN") that are printed in incongruent ink colors (e.g., the word "RED" printed in blue ink), requiring the participant to name the ink color while ignoring the written word. This task places significant demands on selective attention, cognitive control, and executive function, as it requires overcoming automatic word-reading tendencies to focus on the less automatic color-naming process. The interference effect observed in the Stroop test is thought to be a robust measure of intergroup differences in cognitive control and information processing efficiency [27].

Frontal midline theta and cognitive control

Cognitive interference tasks like the Stroop color-word test are therefore classic paradigms for probing executive attention and conflict resolution in the brain. Successfully resolving Stroop interference, naming the ink color of an incongruent color-word, appears to engage the anterior cingulate cortex (ACC) and a fronto-parietal control network that monitors and resolves response conflict [28]. In the present study, we identified a fronto-parietal midline axis operating in medical students subjected to Stroop testing. We found lower fronto-central than parietal alpha voltage amplitudes, suggesting greater fronto-central cognitive trait-like neural signaling. This interpretation results from our hypothesis that trait-like markers would manifest as site-sensitive rather than load-sensitive changes. A neural signature of this process is reported for increased midline theta voltage thought to be indicative of ACC-driven neural oscillations [29]. EEG studies consistently report enhanced midfrontal theta oscillations in the Stroop task and similar cognitive interference paradigms [30]. Such frontal midline theta is thought to be an indicator of conflict detection and the engagement of cortical cognitive control [31].​ This finding is consistent with the notion that frontal theta is a key indicator of cognitive control [32]. In the present study, we also found trait-like behavior in the higher fronto-central than parietal θ/β voltage amplitude ratios. This finding suggests that θ/β ratio may provide a parietal-posterior dominant cognitive attentional control marker for Stroop color-conflict testing.

Beta oscillations and cognitive conflict

Beta oscillations appear to behave inversely to theta oscillations during interference challenges. Beta oscillations are generally associated with an alert, engaged brain state and motor readiness, and they tend to suppress or desynchronize when inhibitory control is required [33]. For example, successful response inhibition in a Go/NoGo task produces a marked reduction in beta power reflecting the interruption of status-quo neural rhythms [34]. In the context of Stroop tasks, evidence is emerging that beta brainwaves may also carry information about conflict processing. A spatial Stroop study demonstrates that in interference trials, beta power changes are correlated with response conflict [35]. Time-frequency analyses of classic Stroop testing show that stronger responses to incongruent stimuli are associated with increased high-beta activity [36]. In addition to the aforementioned parietal dominance of θ/β ratios, favorable for attentional control, high-β/low-β ratios were also lower parietally. These findings suggest that both θ/β and high-β/low-β ratios, found to be insensitive to the Stroop testing cognitive load, qualify as potential cognitive trait biomarkers. The lower high-β/low-β ratio parietally implies less high-β (18-30 Hz) and more low-β (12-18 Hz) activity. This is one of the more interesting findings of our study because this low-β bandwidth overlaps with the 12-15 Hz sensorimotor rhythm (SMR). SMR is known to play a role in several complex cognitive paradigms. For example, healthy adults with larger SMR voltage amplitudes in the parietal-posterior axis were found to be more responsive to audio-visual neurobiofeedback training than those with smaller resting parietal-posterior SMRs [37]. Future studies will, no doubt, build on these findings to determine the extent to which the SMR can be recruited to improve cognitive processing of complex information.

Application of qEEG in student populations

Despite extensive research on the Stroop test, qEEG has, heretofore, not been applied to a cohort of medical students. This cohort is trained to answer complex multiple-choice questions (MCQs) within a strict 90-second time limit, the average period usually needed to earn a passing score on the United States Medical Licensure Examinations (USMLE). In our experience, this requires cognitive skills akin to those needed for Stroop task performance. Answering these MCQs involves: (i) inhibiting distractions (filtering out irrelevant options); (ii) cognitive flexibility (switching between diagnostic possibilities); (iii) rapid information processing (integrating prior knowledge with new clinical data); and (iv) executive control (managing time constraints and stress). These skills align closely with the attentional control, response inhibition, and cognitive interference resolution tested by the Stroop task, suggesting that a qEEG analysis of medical students performing high-stakes MCQs may uncover novel neurophysiological markers of cognitive interference task performance.

Neurophysiological markers of attentional control

Cognitive interference tasks, such as the Stroop color-conflict test, are purported to provide a well-established model for studying goal-directed attentional control, response inhibition, and executive function [38]. The ability to suppress prepotent responses in favor of goal-directed behavior is essential for high-level problem-solving, particularly in medical education, where students must rapidly filter, integrate, and apply clinical information under time constraints [39]. While behavioral Stroop performance has been widely studied, no previous studies have used quantitative qEEG to investigate neurophysiological mechanisms associated with cognitive interference task performance in medical students trained to answer high-stakes MCQs within a strict 90-second time frame.

Theta-beta coupling and cognitive performance

Neural oscillations at different frequencies often interact through cross-frequency coupling to coordinate multifarious brain functions. Such interactions involve phase-amplitude coupling or as co-fluctuations in the amplitudes of different bandwidths [40]. Cross-frequency coupling is believed to facilitate communication between local and distributed neural networks​ [41]. This is implicated in a range of cognitive processes including working memory and attention. Theta-beta coupling appears to play a role in cognition as well. For example, θ-β cross-frequency coupling at midline electrode sites has been associated with the temporal orienting of attention in children [3].

Implications for cognitive trait biomarkers

In the present study, the midline θ/β ratios were identified as a biomarker likely linked to attentional control. Resting qEEG studies show that higher θ/β ratios (excess slow wave theta relative to fast wave beta) are associated with poorer attention and inhibitory control, whereas lower θ/β ratios are linked to better attentional control and cognitive performance [40]. This has been corroborated in pilot studies from our laboratory [42, 43]. Individuals with stronger executive function also tend to exhibit relatively lower θ/β ratios, primarily at frontal sites [40]. This is consistent with the observation that elevated θ/β ratios are characteristic of attention-deficit/hyperactivity disorder (ADHD) and other disorders associated with impairments of attentional control [44].

Broader integration of qEEG markers

Beyond voltage power ratios, researchers have examined direct coupling between slow and fast EEG oscillations. For example, stronger resting delta-beta coupling (positive correlation between delta and beta band amplitudes) at parietal electrodes has been linked to higher self-reported attentional control in healthy adults​ [40]. In the present study, we intentionally omitted delta brainwave analyses because of concerns about stationarity associated with short-term five-minute recording of EEGs. Nevertheless, studies of delta-beta interactions suggest that the brain may be in a more focused and regulated state when slow-wave activity co-varies with fast-wave beta activity. Our study of slow-wave θ interactions with fast-wave β activity in the present was restricted to computing θ/β ratios, as we also reported in similar cohorts of medical students [42, 43].

Toward trait biomarkers for cognitive interference

Previous studies have shown that cognitive load is a multidimensional construct, and different brainwave frequencies may capture various aspects of cognitive effort, task complexity, and mental fatigue​ [45-47]. Thus, integrating multiple qEEG markers could provide a comprehensive picture of how cognitive load fluctuates during learning tasks [48-51]. This study is the first to employ multiple qEEG markers as measures of cognitive skill before and after a series of congruent and incongruent Stroop color-conflict cognitive-interference tasks in medical students.

We found qEEG parameters that differed by scalp site and resisted modulation by transient cognitive-load changes within a single Stroop testing session. We believe these results represent a first step toward identifying features for biomarkers of inefficient information processing under a condition of examination stress-inducing test anxiety in susceptible individuals.

Summary of key findings

In summary, alpha voltage amplitude reproduced classic posterior > frontal gradients, whereas θ/β and high-β/low-β ratios were lowest parietally, mirroring the topography of known vigilance networks [52]. These patterns persisted through the LL and HL Stroop tests, thereby meeting our criterion for alpha voltage amplitude, θ/β, and high-β/low-β ratios as candidates for biomarkers of cognitive information processing. However, the cognitive load insensitivity of these biomarkers suggests that alpha voltage amplitude θ/β, and high-β/low-β ratios may not be useful for monitoring cognitive effort. Such cognitive load-insensitive biomarkers are thought to be reflected in thalamocortical pacemaker activity [53] and align with findings that θ/β ratio is an indicator of test anxiety and executive effort in ADHD patients [54, 55].

Neural coordination of cognitive control

Results of the present study appear to support the findings of a meta-analysis indicating that qEEG measures of cognitive workload may be frequency- and site-specific [56]. These findings also corroborate previous research implicating the parietal lobe as a critical hub for Stroop color-conflict cognitive-interference task performance [57-59]. Co-modulation of theta and beta activity has been interpreted as a marker for top-down cognitive control processes and the continuous integration of goal-directed (beta) and conflict-monitoring or error-detection (theta) signals [60-62]. Theta oscillations, typically originating in frontal midline structures, may play an important role in the detection of cognitive conflict and the initiation of executive control, while beta-band dynamics have been linked to the maintenance of task-relevant rules and motor output [63, 64]. Physiologically, this co-transmission of theta and beta is thought to facilitate the swift transition between conflict detection (reflected in theta bursts) and task execution (supported by beta synchronization) as the brain’s neural networks strive to maintain performance accuracy [65]. These findings further suggest that qEEG may capture subtle variations in theta and beta amplitudes which could be useful for observing real-time coordination of neural processes associated with Stroop testing [66, 67].

Educational implications

Embedding quick qEEG screens early in medical curricula could flag students whose qEEG profiles predict MCQ-related cognitive strain. Coupling this with θ-β biofeedback [68] may enhance working-memory resilience before high-stakes tests.

Limitations

The present investigation should be regarded as preliminary and exploratory. Although the protocol extends qEEG methodology to a rarely studied population of medical students, several factors constrain the strength and generalizability of the conclusions. Because of the small sample size and outlier removal, the study was powered to detect large effects; smaller but potentially significant effects may have gone undetected. Consequently, point estimates of effect size and correlation should be considered tentative until replicated in a larger cohort. Recordings were obtained solely from the midline sites Fz, Cz and Pz. While these positions measure activity of the medial fronto-parietal network, they do not sample lateral pre-frontal, temporal or parietal regions that may be more sensitive to cognitive load. Therefore, the absence of load-related changes at Fz, Cz and Pz cannot be taken as evidence of a null effect; future high-density montages studies should map the full topography of task-induced qEEG changes. The stability of the Fz/Cz/Pz amplitudes across load levels raises the possibility that these midline may measure or index relatively enduring cognitive functions (e.g., working-memory capacity, cognitive control style) rather than transient task engagement. Clarifying this distinction will need longitudinal testing and correlation with independent assessments of cognitive skills. Such studies could employ an array of psychometric tests to validate the qEEG measures.

Participants were drawn from a narrowly defined group of medical students. Physiological adaptations unique to this population may limit the applicability of the findings to more diverse samples in age, sex, fitness level or clinical status. All recordings were collected in one session, precluding assessment of intra-individual stability, learning effects or day-to-day variability. Multi-session or longitudinal designs will be necessary to establish reliability and causal inferences. In view of these limitations, the present results should be interpreted as hypothesis-generating rather than definitive. Replication with larger, more heterogeneous samples and full-head, high-density qEEG together with longitudinal follow-up is likely to confirm whether mid-line RMS amplitudes constitute reliable markers of individual cognitive abilities or merely reflect sampling constraints of the current design. The main limitation of the present study is, therefore, the small sample and strict outlier removal, which, no doubt, decreased the study’s statistical power. Replication with more detailed analysis of the biomarkers at the recording sites lateral to the midline sites employed in this study, together with behavioral correlates such as anxiety scores, should improve the study’s sensitivity.

Finally, this pilot study was designed to identify qEEG markers most likely to be robust across a broad population of medical students, regardless of underlying medical, psychological, or sociodemographic status. Our goal was to establish foundational parameters that could eventually support future, more tightly controlled investigations across diverse student populations exposed to the cognitive demands of high-stakes, time-limited examinations. Factors such as IQ, personality traits, psychiatric history, and medical comorbidities may influence EEG responses and cognitive load. However, controlling for these variables was beyond the scope of this exploratory study, which aimed to maximize electroencephalographic validity rather than restrict variability. Future confirmatory studies should incorporate these parameters either through exclusion criteria or statistical control to refine the specificity and interpretability of the findings.

## Conclusions

The results of this study suggest that medical students trained to answer complex, timed multiple-choice questions showed changes in their brain activity during Stroop testing. The alpha brainwave dominant frequency changed with mental workload but didn’t vary by brain region, while other measures such as alpha voltage amplitude, the θ/β and the high-β/low-β ratios, varied by brain region but not with workload. These results suggest that qEEG measurements may help identify specific brain regions involved in task-specific responses, while changes in alpha frequency may help monitor how hard the brain is working. Since this was a small, early study, the results should be viewed as preliminary until confirmed by larger studies in other groups of students preparing for timed, high-stakes exams.
